# Content validation of Mental Health Literacy Scale (MHLS) for primary health care workers in South Africa and Zambia ─ a heterogeneous expert panel method

**DOI:** 10.1080/16549716.2019.1668215

**Published:** 2019-11-08

**Authors:** Joonas Korhonen, Anna Axelin, Gerhard Grobler, Mari Lahti

**Affiliations:** a Faculty of Health and Well-Being, Turku University of Applied Science, Turku, Finland; b Department of Nursing Science, University of Turku, Turku, Finland; c Department of Psychiatry, School of Medicine, University of Pretoria, Pretoria, South Africa

**Keywords:** Health literacy, stigma, cultural validity, mental health disorder, nurse, developing country, knowledge, attitude

## Abstract

**Background**: The lack of public knowledge and the burden caused by mental-health issues’ effect on developing and implementing adequate mental-health care for young and adolescent in low- and middle-income countries (LMIC). Primary health care could be the key in facing the challenge, but it suffers from insufficient resources and poor mental health literacy. This study’s aim was to adapt the content validity of the Mental Health Literacy Scale (MHLS) developed by O’Connor & Casey (2015) with researchers and primary health-care workers in low- and middle-income contexts in South Africa (SA) and in Zambia.

**Objectives**: The study population comprised two expert panels (N = 21); Clinical Experts (CE) (n = 10) from Lusaka, Zambia and Professional Research Experts (PE) (n = 11) from the MEGA project management team were recruited to the study.

**Methods**: MHLS was validated in a South African and a Zambian context using a heterogeneous expert-panel method. Participants were asked to rate the 35 MHLS items on a 4-point scale with 1 as not relevant and 4 as very relevant After the rating, all 35 MHLS items were carefully discussed by the expert panel and evaluated according their relevance. The data were analyzed using an item-level content validity index (I-CVI) and narrative and thematic analyses.

**Results**: All 35 items ranked by the PREs met the cutoff criteria (≥0.8), and ten (n = 10) items were seen as relevant by CE when calculating I-CVIs. Based on the results of ratings and discussion, a group of sixteen (n = 16) of all items (n = 35) were retained as original without reviewing. A total of nineteen (n = 19) items were reviewed.

**Conclusion**: This study found the MHLS to have sufficient validity in LMICs’ context but also recognized a gap between professional researchers’ and clinical workers’ knowledge and attitudes related to mental health.

## Background

Over 85% of the world’s population live in low- and middle-income countries (LMIC), and represent the great majority (>80%) of people with mental health disorders [1,2] without access to proper treatment [3]. These numbers clearly point out the burden of inadequate mental health care especially in LMIC countries. One main barrier for adequate care is the lack of trained health-care professionals; the World Health Organization (WHO) [4] estimates there is only one trained mental health worker serving more than 200.000 people in LMICs.

Understanding cultural needs in terms of mental health is a core component of health promotion [2,5]. The WHO [6] acknowledges the public’s lack of knowledge about mental disorders in children and in adolescents as one of the main barriers to adequate mental health care worldwide. Considering the cultural context of mental health in LMICs, complementary practitioners and traditional healers are still the number one choice for help [2,7]. A lack of knowledge, stigma related to mental health issues, and missing resources are widely recognized barriers to implementing and accessing mental health care [2,5,8].

On a policy level, mental health care’s integration into primary care is recommended [4,7]. According to Atilola [5], training community health workers on mental health and psychiatric care topics would also greatly benefit mental health education programs. Thus, psychiatric nurses [9] and primary health-care workers [5] would have a key role in providing mental health treatment and services.

With mental-health services already scarce in developing countries, poor mental health literacy among primary health-care professionals contributes to the disease’s burden and the indequate treatment of those in need [7]. The concept of ‘mental health literacy’ can be defined as ‘knowledge and beliefs about mental disorders which aid their recognition, management or prevention’ [10]. Previous studies have shown that training health-care workers effectively improves knowledge in mental health literacy [7,11,12], which relieves the burden of mental health disorders [7].

With mental health literacy (MHL), the recognition, knowledge and attitudes [13] toward mental health can be assessed from a professional perspective [12,14,15]. Previous studies lacked an assessment of all MHL attributes, including recognition, knowledge and attitudes towards mental health [13,14]. The new scale-based measure, the Mental Health Literacy Scale (MHLS) includes all MHL attributes. It was originally developed and tested in Australia by O’Connor & Casey [14]. The scale has demonstrated excellent methodological quality in psychometrics for internal consistency, content and structural validity [16] and internal and test–retest reliability [14].

This study aimed to adapt the content validity [17] of the MHLS with researchers and primary health care workers in low- and middle-income contexts in South Africa (SA) and in Zambia.

## Methods

### Study design

In this study, MHLS was validated in SA and in Zambia using a heterogeneous expert-panel method [17]. Research experts and clinical experts evaluated the instrument’s content validity in two phases from April to May in 2018.

The study is a part of a larger European Union-funded project: ‘MEGA–Building capacity by implementing mhGAP mobile intervention in SADC countries’ (funding number 585,827-EPP-1-2017-1-FI-EPPKA2-CBHE-JP) [18]. In the MEGA project, primary health-care workers will be trained to screen young and adolescents’ mental health problems using a new mobile application.

### Study setting and population

The study population comprised two expert panels (N = 21) divided into clinical experts (primary health-care workers) and professional research experts (MEGA project researchers) [17]. Ten clinical experts (CEs) (n = 10) from a primary health clinic in Lusaka, Zambia were recruited by The University of Zambia in April, 2018. The CEs were invited to secure a cultural understanding of mental health in low- and middle-income contexts in Zambia. Currently, screening for mental health issues among young and adolescent is over-burdened as five psychiatrists are responsible for approximately 13 million people [19]. Thus, screening and care is provided mainly by specially trained nurses [9,18]. In this study, the participating primary health-care workers had to meet adolescents regularly in their clinical practice. Eleven (n = 11) professional research experts (PREs) from the MEGA project’s management team were recruited to secure a theoretical understanding of mental health literacy. PREs (i.e psychiatrists, psychologists and psychotherapists) had scientific backgrounds and a long-term understanding of young and adolescent mental health in low- and middle-income contexts.

### Data collection

The first expert panel, consisting of PREs, was organized in University of Free State, South Africa in April, 2018. The second expert panel, with the CEs, was held in the local hospital in Lusaka, Zambia in May, 2018. The investigator informed participants on the expert panels via an informative letter. Every participant was asked for a written informed consent prior to the study. Participating in the panel was voluntary. After the informed consent procedure, participants were asked to rate each of the 35 MHLS items on a 4-point scale [17,20] with 1 as ‘not relevant,’ 2 as ‘unable to assess relevance without revision of the item,’ 3 as ‘relevant but needs minor alteration’ and 4 as ‘relevant to the process of measurement of the MHL.’ After the rating, all 35 MHLS items were carefully discussed within the expert panel and evaluated according their relevance. The instrument went through each item and experts could state whether an item is relevant or not or if it needs revising. At the suggestion of the original MHLS author to meet given changes in DSM-5, the terms of the disorders were modified on two scale items before the data collection.

At the beginning of the expert panel, participants were asked for background information of country, working region and experience, age, gender, education and current profession. Both discussions in expert panel were audio recorded for transcription and analyses. The researcher J. Korhonen was responsible for leading discussions, and the other researcher took field notes about the discussion to support analyses [21]. A total of 23 transcribed pages (A4, Times New Roman, font size 12 and single line spacing) consisting of the field notes (8 pages) and three hours of audio recordings were analyzed with narrative and thematic analyses [22,23].

### Data analysis

The data were analyzed using an item-level content validity index (I-CVI) [20,24]. A CVI is commonly used when deciding whether to delete, revise or retain research scale items [20]. I-CVIs for (1) PREs (I-CVI-PRE, n = 11), (2) CEs (I-CVI-CE, n = 10) and (3) the total group of experts (I-CVI-ALL, n = 21) were calculated for each of the instrument’s 35 items. On a 4-point scale, the best overall item score is 1.00. A cutoff point (≥0.8) was used in analyzing the relevance (Schilling et al. 2008). If the I-CVI-PRE of an item met the cutoff of ≥0.8 but I-CVI-ALL did not, the item was evaluated critically, and a decision was made to retain, eliminate or revise the item based on experts’ suggestions and ratings during the expert panel discussions [17]. The item rating (1 to 4) was signed as ‘*’ if it was unclearly marked by the experts. The I-CVI-Clinical was reported separately only for a better understanding of phenomena in the research. The final decision was made according to the PREs’ suggestion. The average CVIs for the entire scale (S-CVI/Ave, 35 items) were calculated for both expert panels with a cutoff criteria of (≥0.9) for entire scales [17,20].

## Results

### Characteristics of participants

In line with the protocol, both PRE and CE experts, totaling 21, participated in the study (N = 21). Eleven (n = 11) of these were PREs, and ten (n = 10) were CEs, representing the target population of the main research. Female and male experts were involved from four (n = 4) different countries. Participants’ backgrounds in professional education varied from a certificate to a PhD. When asked for work titles or current professions, PREs had both clinical and research backgrounds. None of the CEs reported research background in a professional health category. The majority (n = 8) of all PREs reported a work experience of 15 years or more, while half (n = 5) of the 10 CEs reported their working experience as up to 5 years. A full demography of participants is presented in Table 1.

**Table 1.** Demography of participants.

	Professional Research Experts (PREs) (n = 11)	Clinical Experts (CEs) (n = 10)
**Country**		
South Africa	5	-
Zambia	3	10
Germany	2	-
Finland	1	-
**Sex**		
Female	8	8
Male	3	1
(not reported)		1
**Age by group**		
Mean (SD)	48.5 (12.4)	32.5 (8.3)
**Health professional category**		
Registered nurse/midwife		7
Enrolled nurse		1
Psychotherapist	1	
Lecturer	6	
Research nurse	2	
Research psychologist	2	
Clinical officer general		1
Art nurse		1
**Level of professional education**		
Certificate		2
Bachelor/Diploma	2	8
Master’s	4	
PhD	5	
**Work experience**		
Less than 1 year		2
1 to <5 years	1	3
5 to <10 years	1	1
10 to <15 years	1	-
15 years or more	8	4

### Content validity of the MHLS

The item-level content validity index (I-CVI) for all 35 MHLS items was rated by eleven (n = 11) PREs and by ten (n = 10) CEs. The I-CVIs for the 35 items ranked by the PREs varied from 0.82 to 1.00, and the CEs’ rankings ranged from 0.1 to 1.00. Among PREs, all the MHLS items (n = 35) met the cutoff criteria of ≥0.8 for the I-CVI. The average of the I-CVIs (S-CVI/ Ave) for all items on the scale within PREs was 0.95, meeting the desired cutoff criteria (≥0.9). The 35 item ratings by PREs on a 4-point relevance scale (Polit & Beck 2006) are shown in Table 2.

**Table 2.** Ratings for 35 items by PREs: items rated 3 or 4 (x) on a 4-point relevance scale [20].

Item	Expert 1	Expert 2	Expert 3	Expert 4	Expert 5	Expert 6	Expert 7	Expert 8	Expert 9	Expert 10	Expert 11	Number in Agreement	Item CVI (I-CVI-PE)
1	*	x	x	x	x	x	x	x	x	x	–	9	0.90
2	*	x	x	x	x	x	x	x	x	x	x	10	1.00
3	x	x	x	x	x	x	x	x	x	x	x	11	1.00
4	–	x	x	x	x	–	x	x	x	x	x	9	0.82
5	–	x	x	x	x	x	x	x	x	x	–	9	0.82
6	x	x	x	x	x	x	–	x	x	x	x	10	0.91
7	x	x	x	x	x	x	x	x	x	x	x	11	1.00
8	x	x	x	x	x	x	x	x	x	x	x	11	1.00
9	x	x	x	x	x	x	x	–	x	–	x	9	0.82
10	x	x	x	x	x	x	x	–	x	–	x	9	0.82
11	x	x	x	x	x	x	x	x	*	x	x	10	1.00
12	x	x	x	x	x	x	x	–	x	x	x	10	0.91
13	x	x	x	x	x	x	x	x	x	x	x	11	1.00
14	x	x	x	x	x	x	x	x	x	x	x	11	1.00
15	x	x	x	x	x	–	x	x	x	x	x	10	0.91
16	x	x	x	x	x	x	x	x	x	x	x	11	1.00
17	x	x	x	x	x	x	x	x	x	x	x	11	1.00
18	x	x	x	x	x	x	x	x	x	x	x	11	1.00
19	x	x	x	x	x	x	x	x	x	x	x	11	1.00
20	x	x	x	x	x	–	x	x	x	x	x	10	0.91
21	x	x	x	x	x	x	x	x	x	x	x	11	1.00
22	x	x	x	x	x	x	x	x	x	x	x	11	1.00
23	x	x	x	x	x	x	x	x	x	x	x	11	1.00
24	x	x	x	x	x	–	x	x	x	x	x	10	0.91
25	x	x	x	–	x	x	x	x	x	x	x	10	0.91
26	x	x	x	x	x	x	x	x	x	x	x	11	1.00
27	x	x	x	x	x	x	x	x	x	x	x	11	1.00
28	x	x	x	x	x	–	x	x	x	x	x	10	0.91
29	x	x	x	x	x	x	x	–	x	x	x	10	0.91
30	x	x	x	x	x	x	x	–	x	x	x	10	0.91
31	x	x	x	x	x	x	x	x	x	x	x	11	1.00
32	x	x	x	x	x	x	x	x	x	x	x	11	1.00
33	x	x	x	x	x	–	x	x	x	x	x	10	0.91
34	x	x	x	x	x	x	x	x	x	x	x	11	1.00
35	x	x	x	x	x	x	x	x	x	x	x	11	1.00
**Mean Item level CVI (S-CVI/Ave)**													0.95

* = Unclearly marked by the expert

Among CEs, ten (n = 10) items out of all I-CVI items (n = 35) met the cutoff criteria (≥0.8). The average I-CVI (S-CVI/Ave) for all items on the scale rated by CEs was 0.62. When calculating mean validity index for the both expert groups, PREs and CEs of experts, S-CVI/Ave was 0.8. Ratings on 35 items by CEs on a 4-point relevance scale (Polit & Beck 2006) are shown in Table 3.

**Table 3.** Ratings for 35 items by CEs: items rated 3 or 4 (x) on a 4-point relevance scale [20].

Item	Expert 1	Expert 2	Expert 3	Expert 4	Expert 5	Expert 6	Expert 7	Expert 8	Expert 9	Expert 10	Number in Agreement	Item CVI (I-CVI-CE)
1	–	x	x	–	x	–	–	x	–	x	5	0.5
2	–	x	x	–	x	x	–	x	–	x	6	0.6
3	–	x	x	x	x	x	–	x	x	–	7	0.7
4	x	x	–	–	x	x	–	x	–	x	6	0.6
5	*	x	x	x	x	–	x	x	–	–	6	0.67
6	x	x	x	–	–	x	x	x	x	x	8	0.8
7	–	–	x	x	x	x	x	x	–	x	7	0.7
8	x	x	x	x	x	x	x	x	–	–	8	0.8
9	–	–	–	–	–	–	–	–	*	x	1	0.1
10	–	–	–	–	–	–	–	–	x	x	2	0.2
11	x	x	x	–	x	–	x	x	x	x	8	0.8
12	–	–	x	–	–	x	x	x	x	–	5	0.5
13	x	x	x	x	x	x	x	x	x	–	9	0.9
14	x	x	x	–	x	x	x	x	x	x	9	0.9
15	x	x	–	–	x	x	–	x	x	–	6	0.6
16	x	x	x	x	x	x	x	x	x	x	10	1.00
17	x	x	x	x	x	x	–	x	x	x	9	0.9
18	x	x	–	x	x	x	x	x	x	x	9	0.9
19	x	x	x	x	x	x	x	x	x	x	10	1.00
20	x	x	x	x	–	–	–	–	x	–	5	0.5
21	x	–	x	–	x	x	–	–	–	–	4	0.4
22	x	x	x	–	x	x	–	–	–	x	6	0.6
23	x	x	*	x	x	x	x	*	–	–	6	0.75
24	x	–	–	–	x	x	–	–	–	–	3	0.3
25	x	x	–	–	x	–	–	–	x	–	4	0.4
26	x	–	x	–	x	x	–	–	x	–	5	0.5
27	x	x	–	–	x	–	–	–	x	–	4	0.4
28	x	x	–	–	–	x	–	–	–	–	3	0.3
29	x	x	–	–	x	x	x	x	–	x	7	0.7
30	x	x	–	–	x	–	x	x	–	–	5	0.5
31	x	x	x	x	x	–	x	x	x	–	8	0.8
32	x	x	x	–	x	–	x	–	x	–	6	0.6
33	x	x	–	–	x	–	x	–	x	–	5	0.5
34	x	x	–	–	x	x	x	–	x	x	7	0.7
35	x	x	–	–	x	x	x	–	x	x	7	0.7
**Mean Item level CVI (S-CVI/Ave)**												0.62

* = Unclearly marked by the expert

### Suggestions for relevance and clarity of MHLS in expert panel discussions

Expert panel discussions were held after both ratings. In the PREs’ panel (n = 11), twenty-nine (n = 29) out of all (n = 35) items were considered relevant. In four cases, items were considered relevant with minor alteration (n = 4), and two (n = 2) items were considered unclear. In the expert panel discussion for CEs (n = 10), twenty-six (n = 26) items out of all (n = 35) items were seen as relevant, and six (n = 6) were considered relevant with minor alteration. Three (n = 3) items were considered unclear.

Following both expert panels, and based on the results of ratings and discussion, a group of sixteen (n = 16) out of all items (n = 35) were retained as original without review. A total of nineteen (n = 19) items were reviewed. Eleven (n = 11) of these reviewed (n = 19) items were modified, but eight (n = 8) items (4,12,21, 26, 27, 28, 30 and 33) were retained as original after careful consideration by the researchers. Five (n = 5) (4, 12, 27, 28 and 33) of these eight (n = 8) retained items were not modified as there was no specific suggestion by the experts; PREs rated and CEs discussed these items as relevant. For three (21, 26 and 30) of these eight (n = 8) reviewed but retained items, the comments reflected CEs’ attitudes and knowledge toward mental health illnesses rather than the evaluation of the items’ relevance, so they were retained as original. Three (n = 3) items (6,7 and 14) that met the cutoff point of 0.8 (I-CVI/ PRE, I-CVI/ALL) were modified for better conceptual clarity, as suggested during the expert panel discussions. Both groups agreed that the MHLS is relevant overall for measuring mental health literacy among primary health-care workers. The ratings, consensuses and rationales of expert panels for 35 items are shown on Table 4.

**Table 4.** Ratings, consensuses and rationale of expert panels on 35 items.

Original Item	Item level CVI (PREs/ CEs/ ALL)	Consensus focus group (PREs/CEs)	Experts’ suggestions/comments	Re-viewed(x)	Rationale	Form of the item after revision
1. If someone became extremely nervous or anxious in one or more situations with other people (e.g., a party) or performance situations (e.g., presenting at a meeting) in which they were afraid of being evaluated by others and that they would act in a way that was humiliating or feel embarrassed, then to what extent do you think it is likely they have **Social Phobia**	0.90/ 0.50/ 0.70	Relevant/Not clear	One PRE questioned if a responder, a patient or nurse, understands the term ‘social phobia’ in the same context. One CE commented ‘not relevant’ because you can’t be anxious or nervous but overexcited for a party. Two other comments were relevant for social phobia.	x	The term ‘in social gatherings’ was added for clearance	If someone became extremely nervous or anxious in one or more situations with other people (e.g., **in social gatherings)** or performance situations (e.g., presenting at a meeting) in which they were afraid of being evaluated by others and that they would act in a way that was humiliating or feel embarrassed, then to what extent do you think it is likely they have Social Phobia
2. If someone experienced excessive worry about a number of events or activities where this level of concern was not warranted, had difficulty controlling this worry and had physical symptoms such as having tense muscles and feeling fatigued then to what extent do you think it is likely they have **Generalised Anxiety Disorder**	1.00/ 0.60/ 0.80	Relevant/Relevant	No specific suggestions	─	─	Retained as original.
3. If someone experienced a low mood for two or more weeks, had a loss of pleasure or interest in their normal activities and experienced changes in their appetite and sleep then to what extent do you think it is likely they have **Major Depressive Disorder**	1.00/ 0.70/ 0.86	Relevant/Relevant	No specific suggestions	─	─	Retained as original.
4. To what extent do you think it is likely that **Personality Disorders** are a category of mental illness	0.82/ 0.60/ 0.71	Relevant/Relevant	CE commented as relevant	x	─	Retained as original.
5. To what extent do you think it is likely that **Persistent Depressive Disorder** (Dysthymia) is a disorder	0.82/ 0.67/ 0.75	Relevant, but needs minor alteration/ Relevant	PREs: ‘Persistent depressive mood’ would be more correct. CE: The word ‘a disorder’ needs to add ‘mental.’	x	Added the term ‘mental,’ as requested and retained the original term ‘disorder’ as suggested by the instrument’s original author.	To what extent do you think it is likely that Persistent Depressive Disorder (Dysthymia) is a **mental** disorder
6. To what extent do you think it is likely that the diagnosis of **Agoraphobia** includes anxiety about situations where escape may be difficult or embarrassing	0.91/ 0.80/ 0.86	Relevant/ Relevant	PREs: The term agoraphobia can be hard to understand. ‘Do they [nurses] know?’	x	Added the term “e.g (open market place)” for clarity.	To what extent do you think it is likely that the diagnosis of Agoraphobia includes anxiety about situations **(e.g., open market place)** where escape may be difficult or embarrassing
7. To what extent do you think it is likely that the diagnosis of **Bipolar Disorder** includes experiencing periods of elevated (i.e., high) and periods of depressed (i.e., low) mood	1.00/ 0.70/ 0.86	Relevant/ Relevant	PRE: Could add a term, such as ‘extremely’ elevated.	x	Added “extremely elevated” for clarity.	To what extent do you think it is likely that the diagnosis of Bipolar Disorder includes experiencing periods of **extremely elevated** (i.e., high) and periods of depressed (i.e., low) mood
8. To what extent do you think it is likely that the diagnosis of **Substance Abuse Disorder** can include physical and psychological tolerance of the drug (i.e., require more of the drug to get the same effect)	1.00/ 0.80/ 0.90	Relevant/ Relevant	No specific suggestions	─	─	Retained as original
9. To what extent do you think it is likely that in general in Australia, **women are MORE likely to experience a mental illness of any kind compared to men**	0.82/ 0.10/ 0.50	Relevant with minor alternation / Relevant with minor alternation	Clinical experts and some professionals stated that the question is relevant if the location ‘Australia’ would be changed. Some PREs mentioned that there is no epidemiological evidence from Zambia, and that’s why it is probably not relevant in this context. A global perspective would be relevant in absence of an epidemiological study, but specifying a mental illness, such as ‘depression,’ would clear it out.	x	Context changed to a global perspective.	To what extent do you think it is likely that in general **women are MORE likely to experience some mental illnesses compared to men**
10. To what extent do you think it is likely that in general, in Australia, **men are MORE likely to experience an anxiety disorder compared to women**	0.82/ 0.20/ 0.52	Relevant/ Relevant with minor alternation	Clinical experts and some professionals stated that the question is relevant if the location ‘Australia’ was changed or left blank. A PRE stated that in Zambia there is no epidemiological information regarding any mental health illness.	x	─	To what extent do you think it is likely that in general, **men are MORE likely to experience an anxiety disorder compared to women**
11. To what extent do you think it would be helpful for someone to **improve their quality of sleep** if they were having difficulties managing their emotions (e.g., becoming very anxious or depressed)	1.00/ 0.80/ 0.90	Relevant/ Relevant	No specific suggestions	─	─	Retained as original.
12. To what extent do you think it would be helpful for someone to **avoid all activities or situations that made them feel anxious** if they were having difficulties managing their emotions	0.91/ 0.50/ 0.71	Not clear/ Relevant	PREs: Not very clear, can be understood differently and needs minor alterations. Not ‘helpful information.’ No specific suggestion was made by PREs in terms of minor alterations. CE: Commented as relevant.	x	─	Retained as original.
13. To what extent do you think it is likely that **Cognitive Behaviour Therapy (CBT)** is a therapy based on challenging negative thoughts and increasing helpful behaviours	1.00/ 0.90/ 0.95	Relevant/ Relevant	No specific suggestions	─	─	Retained as original.
14. Mental health professionals are bound by confidentiality; however there are certain conditions under which this does not apply. To what extent do you think it is likely that the following is a condition that would allow a mental health professional to **break confidentiality**: *If you are at immediate risk of harm to yourself or others*	1.00/ 0.90/ 0.95	Relevant/ Relevant, but needs minor alteration	CE: The phrase ‘who is in risk of harm’ is not clearclear whether it refers to a patient or a nurse. One said it’s clear. The third said the meaning is more of ‘a professional [nurse].’ The term ‘you’ was substituted with the term ‘patient.’	x	The patient is at immediate risk to oneself or to others.	Mental health professionals are bound by confidentiality; however there are certain conditions under which this does not apply. To what extent do you think it is likely that the following is a condition that would allow a mental health professional to break confidentiality: *If **a patient i**s at immediate risk of harm to oneself or others*
15. Mental health professionals are bound by confidentiality; however there are certain conditions under which this does not apply. To what extent do you think it is likely that the following is a condition that would allow a mental health professional to **break confidentiality**: *if your problem is not life-threatening and they want to assist others to better support you*	0.91/ 0.60/ 0.76	Relevant/ Relevant	As in item 14, the CE commented as relevant.	x	If a patient’s problem is not life-threatening and professionals want to assist others to better support a patient.	Mental health professionals are bound by confidentiality; however there are certain conditions under which this does not apply. To what extent do you think it is likely that the following is a condition that would allow a mental health professional to break confidentiality: *If **patient’s** problem is not life-threatening and professionals want to assist others to better support a patient*
16. I am confident that I know where to seek information about mental illness	1.00/ 1.00/ 1.00	Relevant/ Relevant	No specific suggestions	─	─	Retained as original.
17. I am confident using the computer or telephone to seek information about mental illness	1.00/ 0.90/ 0.95	Relevant/ Relevant	No specific suggestions	─	─	Retained as original.
18. I am confident attending face to face appointments to seek information about mental illness (e.g., seeing the GP)	1.00/ 0.90/ 0.95	Relevant/ Relevant	PREs discussed if these questions are testing knowledge, stigma and attitude, and said ‘all are relevant.’	─	─	Retained as original.
19. I am confident I have access to resources (e.g., GP, internet, friends) that I can use to seek information about mental illness	1.00/ 1.00/ 1.00	Relevant/ Relevant	No specific suggestions	─	─	Retained as original.
20. People with a mental illness could snap out if it if they wanted	0.91/ 0.50/ 0.71	Relevant, with minor alteration/ Relevant, but needs minor alteration	PRE: The meaning of ‘snap out’ isn’t clear. The term needs to be revised so it can’t be understood differently. The consensus is for ‘put themselves together’ instead of original term. CE: ‘Snap out’ refers to so many different mental health problems, so the ‘question is clear.’	x	─	People with a mental illness could **put themselves together** if they wanted
21. A mental illness is a sign of personal weakness	1.00/ 0.40/ 0.71	Relevant/ Relevant, but needs minor alteration	CE: Some of them are, some are not. A word like ‘some’ should be added.	x	─	Retained as original.
22. A mental illness is not a real medical illness	1.00/ 0.60/ 0.81	Relevant/ Relevant	No specific suggestions	─	─	Retained as original.
23. People with a mental illness are dangerous	1.00/0.75/ 0.89	Relevant/ Relevant	No specific suggestions	─	─	Retained as original.
24. It is best to avoid people with a mental illness so that you don’t develop this problem	0.91/ 0.30/ 0.62	Not clear/ Relevant	A PRE commented ‘meaning which problem?’ Common discussions were about the question not being quite clear. PREs discussed what this question is about, such as attitude or something else. They said, ‘I’m okay,’ ‘then it’s better change’ and ‘you don’t develop this problem.’ CE: This is relevant because there is stigma. ‘You can’t develop mental illness.’	x	─	It is best to avoid people with a mental illness so that you don’t **catch their illness**
25. If I had a mental illness I would not tell anyone	0.91/ 0.40/ 0.67	Relevant, with minor alternation/Relevant	PREs discussed the meanings of the words anyone, everyone and no one. The suggestion was to change it to ‘I would tell no one.’	x	─	If I had a mental illness **I would tell no one**
26. Seeing a mental health professional means you are not strong enough to manage your own difficulties	1.00/ 0.50/ 0.76	Relevant/ Not clear	One CE: ‘If you see a professional, it means that you are failing.’ One was unable to assess, and one said ‘not relevant,’ but the others said ‘relevant.’	x	The CE is reflecting personal attitudes rather than evaluating the item.	Retained as original.
27. If I had a mental illness, I would not seek help from a mental health professional	1.00/ 0.40/ 0.71	Relevant/ Relevant	No specific suggestions	x	─	Retained as original.
28. I believe treatment for a mental illness, provided by a mental health professional, would not be effective	0.91/ 0.30/ 0.62	Relevant/ Relevant	No specific suggestions	x	─	Retained as original.
29. How willing would you be to move next door to someone with a mental illness?	0.91/ 0.70/ 0.81	Relevant/ Relevant, but needs minor alteration	CEs: Unable to assess because it depends on what type of mental illness a neighbor has. Considering the ‘stigma,’ it’s relevant or ‘not relevant.’	─	─	Retained as original.
30. How willing would you be to spend an evening socialising with someone with a mental illness?	0.91/ 0.50/ 0.71	Relevant/ Not clear	CE’s: One said it’s ‘not relevant.’ Most said it needs a minor alteration and commented on ‘which type of an illness,’ such as bipolar disorder. One commented that it’s relevant.	x	─	Retained as original.
31. How willing would you be to make friends with someone with a mental illness?	1.00/ 0.80/ 0.90	Relevant/ Relevant	No specific suggestions	─	─	Retained as original.
32. How willing would you be to have someone with a mental illness start working closely with you on a job?	1.00/ 0.60/ 0.81	Relevant/ Relevant	CE: ‘What type of a job?’	─	─	Retained as original.
33. How willing would you be to have someone with a mental illness marry into your family?	0.91/ 0.50/ 0.71	Relevant/ Relevant	Most CEs commented that it is relevant.	x	─	Retained as original.
34. How willing would you be to vote for a politician if you knew they had suffered a mental illness?	1.00/ 0.70/ 0.86	Relevant/ Relevant	PRE: ‘I wonder if this really relevant. Your knowledge or your attitude against psychiatry, it’s a personal thing you cannot change.’ A comment was made about if a nurse’s opinion on this topic is really needed. PREs commented that it’s not a matter of a single question but the whole scoring on the scale. CE: ‘Very relevant.’	─	─	Retained as original.
35. How willing would you be to employ someone if you knew they had a mental illness?	1.00/ 0.70/ 0.86	Relevant/ Relevant	No specific suggestions	─	─	Retained as original.
**Mean I-CVI (S-CVI/Ave)**	0.95/ 0.62/ 0.80					

## Discussion

Our purpose was to explore the content validity [17] of the MHLS developed by O’Connor & Casey [14] in low- and middle-income contexts using a heterogeneous expert panel. Mental-health literacy is widely recognized as a key concept for better mental-health knowledge among researchers [7,11,12]. As stated before, primary health-care workers have a key frontline position to render mental-health service [5], but many of them still have an insufficient understanding of mental health [25,26]. The MHLS has shown excellent methodological validity in previous literature, and this study found that the MHLS also has sufficient validity in low- and middle-income contexts. Nevertheless, a gap between professional research experts’ and clinical experts’ knowledge on mental health was recognized. For the first time, this study explored the content validity of the MHLS in this context with two expert panels.

As discussed in previous studies [17], using a heterogeneous panel with experiential experts and professional research experts is fairly uncommon. By using CEs in this study, the researcher wanted to hear CEs’ voices on improving the context-specific relevance of the MHLS. However, PREs’ and CEs’ opinions differed remarkably in the study. All 35 items ranked by the PREs met the cut-off criteria (≥0.8), but only ten (n = 10) items were seen as relevant by CEs when calculating I-CVIs. This reveals that CEs were not familiar with this method or did not have adequate knowledge to evaluate the MHLS. During the expert panel discussion, however, CEs reached a consensus of relevance (relevant or relevant with minor changes) in 32 out of the 35 total items. In addition, a validity index for the overall scale (S-CVI/Ave) rated by PREs easily met the preferred criterion, but CEs’ mean rating for the scale stayed well below the cutoff line. However, even differences between the two groups were notable. After the expert panel, only eleven (n = 11) MHLS items were modified. As a result of the expert panel discussion, CEs agreed that the MHLS was ‘very relevant’ in measuring mental-health literacy among primary health-care workers in their African region, even if they had challenges in separating their personal opinions regarding mental-health disorders from the purpose of the study.

CEs had more difficulties in separating the rating of the items’ relevance from answering the mental-health questions on the scale itself. During the panel, the CEs were obviously reflecting their own knowledge and attitudes toward mental-health topics. This strengthens previous findings of primary health-care workers’ insufficient mental-health knowledge and negative attitudes toward mental-health issues [25,26]. As an explanatory factor, Kapungwe et al. [26] reported that most of their Zambian study population was young (from 25 to 45 years old). Findings also revealed relatively young ages and work experience within the CE group. The demography showed that the half (=5) of participating CEs (n = 10) had work experience of up to five years, and none had done master’s level studies. However, most PREs had 15 years or more experience in the mental-health field. This finding again supports previous studies [7,9,27] on the need to train primary health-care workers on mental-health issues in LMICS. This also aligns with the country’s Mental Health and Poverty Project (MHaPP) report [28] stating that mental-health workers have not received training in human rights nor any refresher courses in the last five years in Zambia. This should be considered when training the local primary-care workers in the future.

### Strengths and limitations

This study has several methodological limitations that must be considered when interpreting the results. Firstly, the expert panel discussion was key when deciding to retain or revise the MHLS items. During the expert panel discussions, the attitudes of CEs were strongly represented in the mental health topics’ terms. This obviously affected the experts’ decisions and reasoning. Researchers needed sensitivity in understanding an accurate consensus, even if some participants were reflecting their own attitudes or knowledge rather than evaluating the item’s relevance, which was this study’s purpose. For better reliability in interpreting the results, the authors suggest monitoring expert panels with a co-researcher with field notes and audio recordings.

Secondly, the study included a fairly big group of experts in the both groups, which isn’t necessary when using a CVI with the expert panel method [17,20].The more experts involved in study, the more complex total agreement within the experts may become [20]. Using focus groups in the expert panels may especially help to understand the phenomenon as common in explanatory studies, but may also lead to different interpretations with different group sizes. This can partly be avoided by following a suggestion by Polit & Beck [20] to use a more relaxed calculation, such as S-CVI/Ave, for a content-validity index instead of analyzing experts’ individual behaviors.

Finally, this study did not involve re-study phase [20] or explore the effect of revising and modifying MHLS items for content validity. Nevertheless, the final decision of whether to retain the item was based on PREs’ ratings. However, CEs’ suggestions for revising items regarding the relevance of the scale were carefully considered in the expert panel discussions. In the future, there is a need for psychometric testing of the validated MHLS version in LMICs’ contexts.

## Conclusion

Using expert panels can be a useful method to culturally and contextually validate an instrument in LMIC settings. The MHLS seems to hold a strong content validation in South African and Zambian contexts. However, more evidence is needed to show the reliability of the MHLS in the previously mentioned context. Therefore, more research needs to be done among primary health workers to show their level of understanding related to MHL. Currently, studies reporting all the Mental Health Literacy components in LMICs are lacking, especially from a professional perspective. More studies are needed to explore and to fill this gap.

## Paper context

Due to the disease burden and lack of knowledge, improving mental-health literacy (MHL) among primary health-care workers in LMICs is crucial for developing and implementing proper mental health care for young and adolescent. This study found that the Mental Health Literacy Scale has sufficient validity in low- and middle-income contexts, but it also recognized a gap between professional researchers’ and clinical workers’ mental health knowledge and attitudes, which strengthened the need for more research.

## References

[1] Patel V. Mental health in low- and middle-income countries. Br Med Bull. 2007;81–82:81–12. 10.1093/bmb/ldm010 17470476

[2] Rathod S, Pinninti N, Irfan M, et al Mental health service provision in low- and middle-income countries. Health Serv Insights. 2017;28:1–7. 10.1177/1178632917694350 PMC5398308 28469456

[3] Anthes E Mental health: there’s an app for that. Nature. 2016;532:20–23. 27078548 10.1038/532020a

[4] World Health Organization Comprehensive mental health action plan 2013–2020. Geneva: WHO Document Production Services; 2013 [cited 2019 828]. Available from: https://apps.who.int/iris/bitstream/handle/10665/89966/9789241506021_eng.pdf?sequence=1

[5] Atilola O Mental health service utilization in sub-Saharan Africa: is public mental health literacy the problem? Setting the perspectives right. Glob Health Promot. 2006;23:30–37. 10.1177/1757975914567179 25749253

[6] World Health Organization Atlas: child and adolescent mental health resources: global concerns, implications for future. Geneva: World Health organization; 2005 [cited 2019 828]. Available from: https://www.who.int/mental_health/resources/Child_ado_atlas.pdf

[7] Ganasen KA, Parker S, Hugo CJ, et al Mental health literacy: focus on developing countries. Afr J Psychiatry. 2008;11:23–28. 10.4314/ajpsy.v11i1.30251 19582321

[8] Sweetland AC, Oquendo MA, Sidat M, et al Closing the mental health gap in low-income settings by building research capacity: perspectives from mozambique. Ann Glob Health. 2014;80:126–133. 24976551 10.1016/j.aogh.2014.04.014 PMC4109687

[9] Alburquerque-Sendín F, Ferrari AV, Rodrigues-de-Souza DP, et al The experience of being a psychiatric nurse in South Africa: a qualitative systematic review. Nurs Outlook. 2018;66:293–310. 29573827 10.1016/j.outlook.2018.01.002

[10] Jorm AF Mental health literacy. public knowledge and beliefs about mental disorders. Br J Psychiatry. 2000;177:396–401. 11059991 10.1192/bjp.177.5.396

[11] Graham AL, Julian J, Meadows G Improving responses to depression and related disorders: evaluation of a innovative, general, mental health care workers training program. Int J Ment Health Syst. 2010;4:25. 20822546 10.1186/1752-4458-4-25 PMC2944816

[12] Armstrong G, Kermode M, Raja S, et al A mental health training program for community health workers in india: impact on knowledge and attitudes. Int J Ment Health Syst. 2011;5:17. 21819562 10.1186/1752-4458-5-17 PMC3169476

[13] O’Connor M, Casey L, Clough B Measuring mental health literacy–a review of scale-based measures. J Ment Health. 2014;23:197–204. 24785120 10.3109/09638237.2014.910646

[14] O’Connor M, Casey L The mental health literacy scale (MHLS): a new scale-based measure of mental health literacy. Psychiatry Res. 2015;229:511–516. 26228163 10.1016/j.psychres.2015.05.064

[15] Liu W, Li Y, Peng Y Mental health literacy: a cross-cultural study of american and chinese bachelor of nursing students. J Psychiatr Ment Health Nurs. 2018;25:96–107. 29139185 10.1111/jpm.12442

[16] Wei Y, McGrath PJ, Hayden J, et al Measurement properties of tools measuring mental health knowledge: a systematic review. BMC Psychiatry. 2016;16:5. 27553955 10.1186/s12888-016-1012-5 PMC4995619

[17] Schilling LS, Dixon JK, Knafl KA, et al Determining content validity of a self-report instrument for adolescents using a heterogeneous expert panel. Nurs Res. 2007;56:361–366. 17846558 10.1097/01.NNR.0000289505.30037.91

[18] Lahti M, Groen G, Mwape L, et al Design and development process of a youth depression screening m-Health application for primary health care workers in South Africa and Zambia: an overview of the MEGA project. Issues Ment Health Nurs. 2019;21:1–7. 10.1080/01612840.2019.1604919 31225763

[19] De Kock JH, Pillay BJ A situation analysis of psychiatrists in South Africa’s rural primary healthcare settings. AOSIS. 2017 [cited 2019 828]. Available from: https://www.ncbi.nlm.nih.gov/pubmed/28582989 10.4102/phcfm.v9i1.1335 PMC5458564 28582989

[20] Polit DF, Beck CT The content validity index: are you sure you know what’s being reported? critique and recommendations. Res Nurs Health. 2006;29:489–497. 16977646 10.1002/nur.20147

[21] Phillippi J, Lauderdale J A guide to field notes for qualitative research: context and conversation. Qual Health Res. 2018;28:381–388. 29298584 10.1177/1049732317697102

[22] Vaismoradi M, Turunen H, Bondas T Content analysis and thematic analysis: implications for conducting a qualitative descriptive study. Nurs Health Sci. 2013;15:398–405. 23480423 10.1111/nhs.12048

[23] Holloway I, Galvin K Narrative inquiry. Qualitative research in nursing and healthcare. Hoboken: John Wiley & Sons, Incorporated; 2016 p. 199–218.

[24] Polit DF, Beck CT Essentials of nursing research: appraising evidence for nursing practice. 9th ed. International ed. Philadelphia: Wolters Kluwer; 2018 p. 78–85, 250.

[25] Mwape L, Sikwese A, Kapungwe A, et al Integrating mental health into primary health care in Zambia: a care provider’s perspective. BMC. 2010;4:21. 10.1186/1752-4458-4-21 PMC2919445 20653981

[26] Kapungwe A, Cooper S, Mayeya J, et al Mental health and poverty project research programme consortium. Attitudes of primary health care providers towards people with mental illness: evidence from two districts in zambia. Afr J Psychiatry. 2011;14:290–297. 10.4314/ajpsy.v14i4.6 22038427

[27] Marais DL, Petersen I Health system governance to support integrated mental health care in south africa: challenges and opportunities. Int J Ment Health Syst. 2015;9:z. 10.1186/s13033-015-0004-z PMC4372271 25806085

[28] Mwanza J, Sikwese A, Mwanza B, et al Country report. Mental health policy development and implementation in Zambia: a situation analysis. Mental Health & Poverty Project (MHaPP). World Health Organization; 2008 [cited 2019 828]. Available from: https://www.who.int/mental_health/policy/development/Zambia%20Country%20report.pdf?ua=1

